# Jian-Pi-Yi-Shen formula alleviates renal fibrosis by restoring NAD+ biosynthesis *in vivo* and *in vitro*

**DOI:** 10.18632/aging.205352

**Published:** 2023-12-28

**Authors:** Liwen Gao, Xi Huang, Ruyu Deng, Shanshan Wu, Yu Peng, Guoliang Xiong, Jiandong Lu, Xinhui Liu

**Affiliations:** 1 Department of Nephrology, Shenzhen Traditional Chinese Medicine Hospital, Guangzhou University of Chinese Medicine, Shenzhen, Guangdong 518033, China; 2 The Fourth Clinical Medical College, Guangzhou University of Chinese Medicine, Shenzhen, Guangdong 518033, China; 3 Shenzhen Traditional Chinese Medicine Hospital Affiliated to Nanjing University of Chinese Medicine, Shenzhen, Guangdong 518033, China

**Keywords:** chronic kidney disease, renal fibrosis, nicotinamide adenine dinucleotide biosynthesis, Jian-Pi-Yi-Shen formula, traditional Chinese medicine

## Abstract

Background: Patients with chronic kidney disease (CKD) lack efficacious treatment. Jian-Pi-Yi-Shen formula (JPYSF) has demonstrated significant clinical efficacy in treating CKD for decades. However, its renoprotective mechanism has not been fully elucidated. This study aimed to determine whether JPYSF could delay renal fibrosis progression in CKD by restoring nicotinamide adenine dinucleotide (NAD+) biosynthesis.

Methods: Adenine-diet feeding was used to model CKD in C57BL/6 mice. JPYSF was orally administered for 4 weeks. Human proximal tubular epithelial cells (HK-2) cells were stimulated with transforming growth factor-β1 (TGF-β1) with or without JPYSF treatment. Renal function of mice was assessed by serum creatinine and blood urea nitrogen levels. Renal histopathological changes were assessed using Periodic acid-Schiff and Masson’s trichrome staining. Cell viability was assessed using a cell counting kit-8 assay. NAD+ concentrations were detected by a NAD+/NADH assay kit. Western blotting, immunohistochemistry, and immunofluorescence were employed to examine fibrosis-related proteins and key NAD+ biosynthesis enzymes expression in the CKD kidney and TGF-β1-induced HK-2 cells.

Results: JPYSF treatment could not only improve renal function and pathological injury but also inhibit renal fibrosis in CKD mice. Additionally, JPYSF reversed fibrotic response in TGF-β1-induced HK-2 cells. Moreover, JPYSF rescued the decreased NAD+ content in CKD mice and TGF-β1-induced HK-2 cells through restoring expression of key enzymes in NAD+ biosynthesis, including quinolinate phosphoribosyltransferase, nicotinamide mononucleotide adenylyltransferase 1, and nicotinamide riboside kinase 1.

Conclusions: JPYSF alleviated renal fibrosis in CKD mice and reversed fibrotic response in TGF-β1-induced HK-2 cells, which may be related to the restoration of NAD+ biosynthesis.

## INTRODUCTION

Chronic kidney disease (CKD), whose prevalence and incidence are on the rise, is defined as the persistent damage of renal function and structure caused by various etiologies for at least 3 months. An epidemiological survey estimated that by 2022, more than 800 million individuals had suffered from CKD, accounting for over 10% of general population worldwide [1]. The poor prognosis and accumulating patients of CKD lay a heavy burden to the international community. However, management of CKD is usually limited to supportive care due to the lack of effective interventions for patients until they need a kidney transplantation or dialysis [2]. Renal fibrosis, characterized by the deposition of extracellular matrix, represents a histopathological hallmark of CKD and an independent risk factor for its progression [3–5]. Therefore, blocking renal fibrosis is a reliable way to delay the progression of CKD. Increasing evidence suggests that traditional Chinese medicine is an efficacious treatment for CKD [6–10]. Jian-Pi-Yi-Shen formula (JPYSF), a Chinese herbal prescription with a decades-long history, has demonstrated significant efficacy in the treatment of CKD. Our previous studies have shown that JPYSF possesses the ability to alleviate renal fibrosis and retard CKD progression in rat models induced by 5/6 nephrectomy or adenine [11–13]. However, the renoprotective mechanism of JPYSF warrants further investigation.

Nicotinamide adenine dinucleotide (NAD^+^) is a vitally indispensable metabolite that exists in all species and plays multiple cellular roles. Initially, NAD^+^ was discovered as an electron carrier participating in a series of redox reactions in cytosol and mitochondria. To be specific, NAD^+^ acts as a co-factor accepting electrons to generate its reduced form NADH, which in turn loses electrons to regenerate NAD^+^. The recycling of NAD^+^-NADH is essential for the uninterrupted flow of electrons throughout redox reactions, encompassing glycolysis, tricarboxylic acid cycle, and fatty acid oxidation (FAO). Without NAD^+^, the cell’s capacity to extract energy in the form of adenosine triphosphate (ATP) from fuel substrates would be impeded [14–16]. Further, studies in the last two decades have found that NAD^+^ also serves as a co-substrate for NAD^+^-consuming enzymes with renoprotective effects including sirtuins [15–20], poly (ADP-ribose) polymerases [14, 15], CD38, and CD157 [14, 21, 22]. In addition, NAD^+^ is a precursor of nicotinamide adenine dinucleotide phosphate (NADP^+^). NADP^+^ and its reduced form, NADPH, play important roles in cellular signal pathways, biosynthetic pathways and antioxidant defence through redox reactions [14, 23]. Therefore, NAD^+^ is not only an indispensable co-factor for energy harvesting but also plays a central role in multiple key molecular mechanisms.

As one of the most active NAD^+^ metabolic organs, kidney requires a high level of NAD^+^ to maintain normal physiological functions, and declined NAD^+^ level has been found in both acute kidney injury (AKI) and CKD [14, 22, 23]. Theoretically, supplementation with exogenous NAD^+^ precursors have a beneficial effect in attenuating disease progression. Indeed, a large body of evidence indicates that augmenting NAD^+^ confers benefits for AKI [22, 24, 25]. However, conflicting results exist in case of CKD [15, 22, 25], which prompted us to concentrate on the enzymes involved in NAD^+^ biosynthesis. The half-life of NAD^+^ is only 1–2 h in the cytoplasm and nucleus and ~8 h in mitochondria [14, 26]. Despite its short half-life time and the multiple reactions consuming it, the intracellular concentration of NAD^+^ in healthy state always changes little, which may attribute to the multiple pathways of NAD^+^ biosynthesis. In mammals, NAD^+^ can be synthesized from three main biosynthesis pathways as follows: (i) the *de novo* pathway from tryptophan; (ii) the salvage pathway including the conversion from nicotinamide (NAM) to nicotinamide mononucleotide (NMN) and nicotinamide riboside (NR) to NMN; (iii) the Preiss-Handler pathway from nicotinic acid (NA) [15, 27]. All enzymes involved in the three biosynthetic pathways described above are expressed in the kidney [16], and some key enzymes have been found to be down-regulated in CKD [28]. We wondered whether key enzymes of NAD^+^ biosynthesis are targets of JPYSF to ameliorate renal fibrosis in CKD. To answer this question, we tested fibrosis, NAD^+^ content, and expression of key enzymes for NAD^+^ biosynthesis in adenine-induced CKD mice and transforming growth factor-β1 (TGF-β1)-stimulated proximal tubular epithelial cells with or without JPYSF treatment.

## MATERIALS AND METHODS

### Antibodies

The primary antibodies against fibronectin (FN, ab2413), type IV collagen (Col-IV, ab6586), quinolinate phosphoribosyltransferase (QPRT, ab171944, for cell Western blotting), nicotinamide mononucleotide adenylyltransferase 1 (NMNAT1, ab45652, for kidney tissue Western blotting and immunofluorescence staining) and nicotinamide riboside kinase 1 (NRK1, ab169548, for cell Western blotting) were purchased from Abcam (Cambridge, MA, USA). Antibodies of anti-vimentin (#5741), anti-α-Tubulin (#3873) and horseradish peroxidase (HRP)-conjugated anti-rabbit IgG (#7074) were obtained from Cell Signaling Technology (Beverly, MA, USA). Antibodies against α-smooth muscle actin (α-SMA, A5228), β-actin (A5441) and QPRT (SAB1410425, for kidney tissue Western blotting and immunofluorescence staining) were from Sigma-Aldrich (St Louis, MO, USA). The anti-NMNAT1 (sc-271557, for cell Western blotting) and anti-NRK1 (sc-398852, for kidney tissue Western blotting and immunofluorescence staining) were from Santa Cruz Biotechnology (Santa Cruz, CA, USA). Antibodies against nicotinic acid phosphoribosyltransferase 1 (NAPRT1, 13549-1-AP), nicotinamide phosphoribosyltransferase (NAMPT, 11776-1-AP), glyceraldehyde-3-phosphate dehydrogenase (GAPDH, 60004-1-IG) and HRP-conjugated anti-mouse IgG (SA00001-1) were purchased from Proteintech Group (Wuhan, China).

### Preparation of JPYSF and identification of major chemical constituents

The raw herbs were procured from Shenzhen Huahui Pharmaceutical Co., Ltd. (Shenzhen, China) and the herbal composition and proportion of JPYSF are shown in Table 1. The mixed crude herbs were soaked in distilled water at room temperature (25°C) for 2 h, and the first decoction was regurgitated with tenfold water (1:10, w/v) for 1.5 h before filtration. Drug residues described above were refluxed with eight times the volume of water (1:8, w/v) for the second decoction and subsequently filtered. The two decoctions were then combined and concentrated. The solution was filtered through a 0.22 μm filter prior to its application onto cultured cells. Identification of major chemical constituents of the extract was analyzed by UPLC-MS/MS and the detailed conditions were described in the Supplementary Materials.

**Table 1:** The herbal composition and proportion of JPYSF.

**Chinese name**	**Botanical name**	**English name**	**Parts used**	**Proportion (g)**
Huang-Qi	Astragalus membranaceus (Fisch). Bge. var. mongholicus (Bge). Hsiao	Astragali Radix	Roots	30 g
Bai-Zhu	Atractylodes macrocephala Koidz.	Atractylodis Macrocephalae Rhizoma	Rhizomes	10 g
Shan-Yao	Dioscorea opposita Thunb.	Dioscoreae Rhizoma	Rhizomes	30 g
Rou-Cong-Rong	Cistanche deserticola Y.C. Ma	Cistanches Herba	Herbs	10 g
Dou-Kou	Amomum kravanh Pierre ex Gagnep.	Amomi Fructus Rotundus	Fruits	10 g
Dan-Shen	Salvia miltiorrhiza Bunge.	Salviae Miltiorrhizae Radix et Rhizoma	Roots and Rhizomes	15 g
Da-Huang	Rheum palmatum L.	Rhei Radix et Rhizoma	Roots and Rhizomes	10 g
Zhi-Gan-Cao	Glycyrrhiza uralensis Fisch.	Glycyrrhizae Radix et Rhizoma Praeparata cum Melle	Roots and Rhizomes	6 g

### Animals and administration

Animal experiments were performed with 8-week-old male C57BL/6 mice (weight: 20 ± 2 g) purchased from Guangdong Medical Laboratory Animal Center (Foshan, China), with the animal certification no. SCXK(YUE) 2018-0002. The animal experiments were outsourced to Shenzhen Top Biotech Co., Ltd. and ethical approval was obtained (approval ID: TOP-IACUC-2022-0108). Prior to the start of experiments, animals were acclimated to the laboratory conditions on a 12-h light/12-h dark cycle at room temperature of 25°C. All mice had free access to food and water. After acclimatization, the mice were randomly assigned to three groups (*n* = 6 mice per group), viz. (1) control group; (2) CKD group (CKD); (3) JPYSF treatment group (CKD+JPYSF). The CKD mouse model was prepared by feeding a diet supplemented with 0.2% w/w adenine for 4 weeks [29]. With simultaneous adenine-diet feeding, mice in the CKD + JPYSF group were gavaged with JPYSF at the dose of 18.3 g/kg/d (JPYSF was administered at a normal dose corresponding to its clinical dose, calculated by the equation: Dose = 121 g/60 kg × 9.1 = 18.3 g/kg/d) [30, 31]. The control group was given normal adenine-free diet. At the end of the 4-week intervention, all the mice were sacrificed and blood samples were obtained; both kidneys were rapidly collected and then a portion of kidney tissue was soaked in 4% paraformaldehyde, embedded in paraffin and sectioned, and the rest of kidney tissue was frozen in liquid nitrogen for further analysis.

### Serum biochemical analysis

Subsequent to blood collection, serum samples were obtained by centrifuging blood for 10 min at 2,000 rpm. Renal function parameters were assessed using commercial kits for the detection of blood urea nitrogen (BUN) and serum creatinine (Scr) (StressMarq Biosciences, British Columbia, Canada).

### Histopathological analysis

After deparaffinization, periodic acid-Schiff (PAS) staining and Masson’s trichrome staining were performed on kidney tissue sections to evaluate renal pathological damage and demonstrate collagen deposition, respectively. In PAS staining, the severity of cortical tubular injury according to tubular atrophy, tubular dilatation, vacuolization, and the degeneration and exfoliation of tubular epithelial cells was scored as followed: 0 = none; 1 = <10% tubules injured; 2 = 10–25% tubules injured; 3 = 26–50% tubules injured; 4 = 51–75% tubules injured; and 5 = >75% tubules injured [32]. The extent of collagen deposition in the kidney was assessed by Masson’s trichrome staining. Areas of positive staining were quantitated by computer-based morphometric analysis and expressed as collagen volume fraction (CVF): CVF (%) = collagen area/total area × 100% using Image J software (NIH, Bethesda, MD, USA). All images of morphological changes were captured under Axio Imager M2 microscope (Carl Zeiss, Jena, Germany). Three microscopic fields (×200) of each mouse and three mice in each group were performed tubular injury score and fibrotic area assessment in a blinded manner.

### Immunohistochemical analysis

Paraffin-embedded kidney sections were progressively processed by deparaffinization, rehydration and antigen retrieval. After incubating with 3% hydrogen peroxide for 10 min at room temperature, the sections were blocked with 10% serum of goat for 1 h at 37°C. Subsequently, the sections were stained with FN, Col-IV, α-SMA and vimentin primary antibody at 4°C overnight. After washing with phosphate buffer saline, the biotin-conjugated secondary antibodies were applied. And then the sections were developed with SignalStain diaminobenzidine (DAB) substrate (Cell Signaling Technology), followed by hematoxylin counterstaining and mounting. The Image J software was utilized to quantify the integrated optical density (IOD) values of positively stained regions. For quantitative analysis, three mice were randomly selected from each group, and three microscopic fields (×200) were observed for each mouse.

### Measurement of NAD^+^

A NAD^+^/NADH assay kit (Beyotime, Nantong, China) was used to determine NAD^+^ levels, according to the manufacture’s instruction.

### Western blotting

Equal amounts of protein were loaded onto 7% or 10% SDS-PAGE gels and subjected to electrophoresis. The separate proteins in SDS-PAGE gels were transferred onto nitrocellulose membranes (Millipore, Billerica, MA, USA) and then subjected to blocking. The membranes were incubated with primary antibodies at 4°C overnight. Subsequently, the membranes were incubated with corresponding secondary antibodies for 1 h at room temperature. Finally, blots were detected and quantification of the bands was performed by measuring the signal gray intensity using the Image Lab™ software (Bio-Rad Laboratories, Hercules, CA, USA).

### Cell culture and intervention

The human renal proximal tubular epithelial cells (HK-2), purchased from Procell Life Science and Technology (Wuhan, China), were cultured in Dulbecco’s Modified Eagle Medium (DMEM; Gibco, Grand Island, NY, USA) supplemented with 10% fetal bovine serum (Gibco), 100 U/mL penicillin, and 100 μg/mL streptomycin. The culture was maintained in a humidified atmosphere at 5% CO_2_ and 37°C. Cells were grown to 80% confluence and treated with different concentrations (0, 2, 5, and 10 ng/mL) of TGF-β1 (PreProtech, Rocky Hill, NJ, USA) for 48 h. To test the protective effect of JPYSF, cells were then incubated with TGF-β1 (10 ng/mL) or/and JPYSF (0.5 and 1 mg/mL) for 48 h.

### Cell viability assay

After exposing with JPYSF at specific concentrations of 0, 0.5, and 1 mg/mL for 48 h, cell viability was determined with a cell-counting kit-8 (CCK-8, Dojindo, Kumamoto, Japan) strictly as per the manufacturer’s manual.

### Immunofluorescence staining

For kidney tissue, the paraffin-embedded sections were processed by deparaffinization, rehydration, antigen retrieval and blocking. Thereafter, the sections were incubated with anti-QPRT, anti-NAPRT1, anti-NMNAT1 and anti-NRK1 overnight at 4°C and subsequently incubated with appropriate secondary antibodies. The fluorescent dye 4′,6-diamidino-2-Phenylindole (DAPI) was used to counterstained nuclei. After 48 h of intervention, the cells were fixed with 4% paraformaldehyde for 20 min, permeabilized with 1% Triton X-100 for 10 min and blocked with 3% bovine serum albumin (BSA) for 1 h at room temperature. The primary antibodies against α-SMA and vimentin were applied and incubated in a humid chamber at 4°C overnight. Finally, the cells were treated with secondary antibodies (Alexa Fluor 488) and DAPI. All slides of kidney tissue and cells were photographed by a fluorescence microscope (Carl Zeiss).

### Statistical analysis

Statistical analysis was conducted using GraphPad Prism software (Version 9; La Jolla, CA, USA). One way analysis of variance (ANOVA) was used to determine significant differences among the groups. All results were presented as mean ± standard error of the mean (SEM). The respective results of *p* value (*p* < 0.05, *p* < 0.01, *p* < 0.001) were all considered statistically significant.

## RESULTS

### Chemical profiling of JPYSF

**Figure 1:**
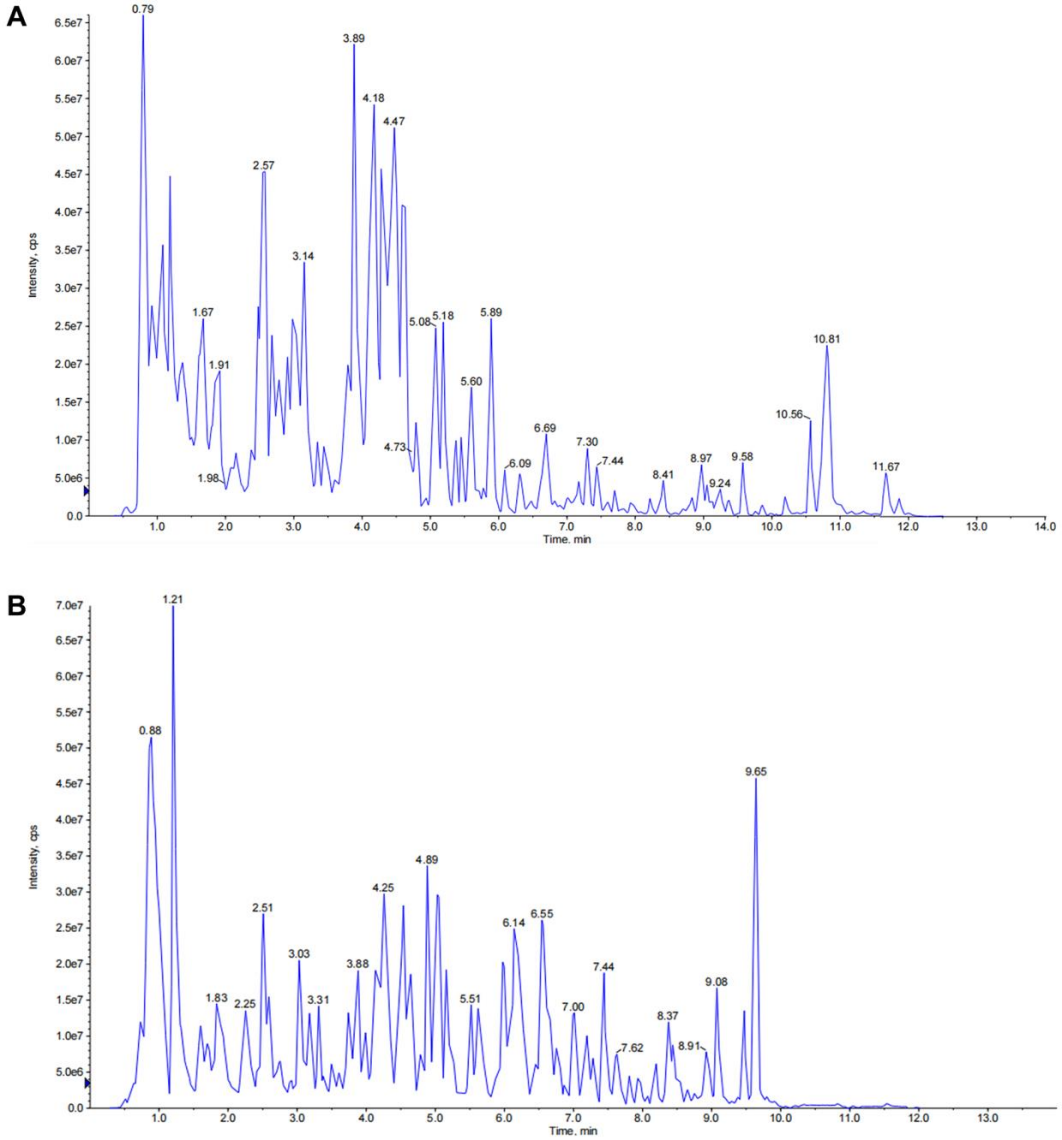
**The total ion chromatograms (TICs) of JPYSF extract.** (**A**) TICs of JPYSF extract in negative ion mode. (**B**) TICs of JPYSF extract in positive ion mode.

The total ion chromatography (TIC) of JPYSF in negative and positive ion modes are presented in Figure 1. In total, 29 constituents were identified including 9 phenolic acids (Cistanoside F, Protocatechualdehyde, Salvianic acid C, Caffeic acid, Vanillic acid, Salvianolic acid G, Salvianolic acid N, salvianolic acid E, and Salvianolic acid A), 8 flavonoids (Isoschaftoside, Baicalin, Isoliquiritin, Daidzein, Quercetin, Calycosin, Genistein, and Glabridin), 8 quinones (Tanshindiol B, Emodin, Neocryptotanshinone, Methyltanshinonate, Epidanshenspiroketallactone, Tanshinone I, Dehydromiltirone, and Miltirone), and 4 terpenoids (Astragaloside II, Astragaloside I, licorice-saponin J2, and Atractylenolide I) (Table 2).

**Table 2:** Chemical constituents identified in JPYSF.

**No.**	**RT (min)**	**Q1 (Da)**	**Q3 (Da)**	**Molecular weight (Da)**	**Formula**	**Ionization model**	**Compounds**	**Class**
1	2.68	487.15	179.03	488.153	C_21_H_28_O_13_	[M-H]−	Cistanoside F	Phenolic acids
2	3.09	139.04	65.04	138.0317	C_7_H_6_O_3_	[M+H]+	Protocatechualdehyde	Phenolic acids
3	3.2	377.09	197.04	378.0956	C_18_H_18_O_9_	[M-H]−	Salvianic acid C	Phenolic acids
4	3.31	179.03	135.05	180.0423	C_9_H_8_O_4_	[M-H]−	Caffeic acid	Phenolic acids
5	3.38	167.03	108.02	168.0423	C_8_H_8_O_4_	[M-H]−	Vanillic acid	Phenolic acids
6	3.38	565.16	409.09	564.1479	C_26_H_28_O_14_	[M+H]+	Isoschaftoside	Flavonoids
7	3.9	417.08	197.02	418.09	C_20_H_18_O_10_	[M-H]−	Salvianolic acid G	Phenolic acids
8	4.23	493.11	313.07	494.1213	C_26_H_22_O_10_	[M-H]−	Salvianolic acid N	Phenolic acids
9	4.28	719.16	323.06	718.1534	C_36_H_30_O_16_	[M+H]+	salvianolic acid E	Phenolic acids
10	4.52	447.09	271.06	446.0849	C_21_H_1_8O_11_	[M+H]+	Baicalin	Flavonoids
11	4.55	419.13	257.08	418.1264	C_21_H_22_O_9_	[M+H]+	Isoliquiritin	Flavonoids
12	4.9	493.11	295.06	494.1213	C_26_H_22_O_10_	[M-H]−	Salvianolic acid A	Phenolic acids
13	4.92	255.07	199.08	254.0579	C_15_H_10_O_4_	[M+H]+	Daidzein	Flavonoids
14	5	303.05	137.02	302.0427	C_15_H_10_O_7_	[M+H]+	Quercetin	Flavonoids
15	5.16	285.08	225.06	284.0685	C_16_H_12_O_5_	[M+H]+	Calycosin	Flavonoids
16	5.37	313.11	249.09	312.0998	C_18_H_16_O_5_	[M+H]+	Tanshindiol B	Quinones
17	5.65	271.06	153.02	270.0528	C_15_H_10_O_5_	[M+H]+	Genistein	Flavonoids
18	6.42	827.48	143.11	826.4715	C_43_H_70_O_15_	[M+H]+	Astragaloside II	Terpenoids
19	7.32	869.49	143.11	868.482	C_45_H_72_O_16_	[M+H]+	Astragaloside I	Terpenoids
20	7.72	825.43	455.35	824.4194	C_42_H_64_O_16_	[M+H]+	licorice-saponin J2	Terpenoids
21	7.93	269.05	225.06	270.0528	C_15_H_10_O_5_	[M-H]−	Emodin	Quinones
22	7.93	325.14	189.09	324.1362	C_20_H_20_O_4_	[M+H]+	Glabridin	Flavonoids
23	8.41	315.16	251.14	314.1518	C_19_H_22_O_4_	[M+H]+	Neocryptotanshinone	Quinones
24	8.54	339.12	205.1	338.1154	C_20_H_18_O_5_	[M+H]+	Methyltanshinonate	Quinones
25	8.69	269.12	195.12	268.1099	C_17_H_16_O_3_	[M+H]+	Epidanshenspiroketallactone	Quinones
26	9.07	277.09	178.08	276.0786	C_18_H_12_O_3_	[M+H]+	Tanshinone I	Quinones
27	9.24	231.14	185.13	230.1307	C_15_H_18_O_2_	[M+H]+	Atractylenolide I	Terpenoids
28	9.79	281.15	253.16	280.1463	C_19_H_20_O_2_	[M+H]+	Dehydromiltirone	Quinones
29	10.21	283.17	268.15	282.162	C_19_H_22_O_2_	[M+H]+	Miltirone	Quinones

### JPYSF improved renal function and pathological injury in CKD mice

**Figure 2:**
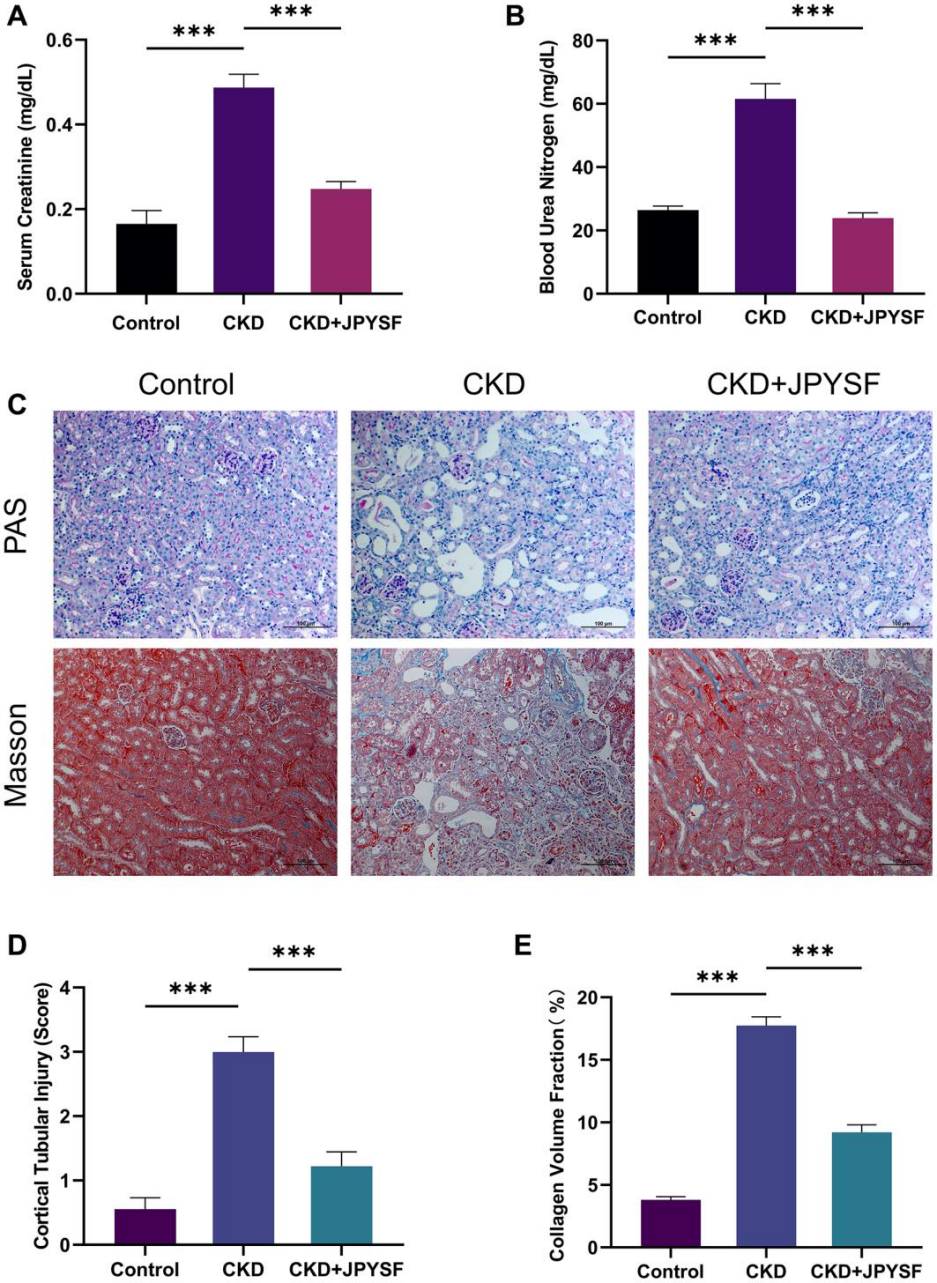
**The effects of JPYSF on renal function and pathological injury in CKD mice.** (**A**) Serum creatinine levels (*n* = 6). (**B**) Blood urea nitrogen levels (*n* = 6). (**C**) Representative PAS and Masson staining images. (**D**) Cortical tubular injury score (*n* = 3). (**E**) Collagen volume fraction (%) (*n* = 3). All images are shown at identical magnification, ×200, scale bar = 100 μm. Data are expressed as mean ± SEM (^***^p < 0.001 between the indicated two groups).

The results of kidney function-related indexes in mice are shown in Figure 2A, 2B. The average levels of Scr and BUN in CKD mice were 2.9 and 2.3 times higher than control mice, respectively (*p* < 0.001), indicating that the CKD mouse model was successfully established. Compared with the CKD group, the levels of Scr (0.25 ± 0.02 mg/dL vs. 0.49 ± 0.03 mg/dL, *p* < 0.001) and BUN (23.90 ± 1.68 mg/dL vs. 61.54 ± 4.85 mg/dL, *p* < 0.001) were markedly reduced in the JPYSF treatment group. In PAS staining, sections from the control group showed no obvious histopathological changes as revealed by clear renal cortical structure and closely arranged renal tubules. In contrast, prominent tubular atrophy, tubular dilatation and vacuolization accompanied by flattening and exfoliation of epithelial cells were observed in the CKD group (Figure 2C, 2D). Masson staining displayed large amount of collagen deposition in the kidney of CKD mice (Figure 2C, 2E). Treatment with JPYSF improved tubular injury and reduced collagen deposition in the kidney of CKD mice (Figure 2C–2E). Taken together, the renal function and histopathological injury could be significantly improved by JPYSF in CKD mice.

### JPYSF suppressed renal fibrosis in CKD mice

**Figure 3:**
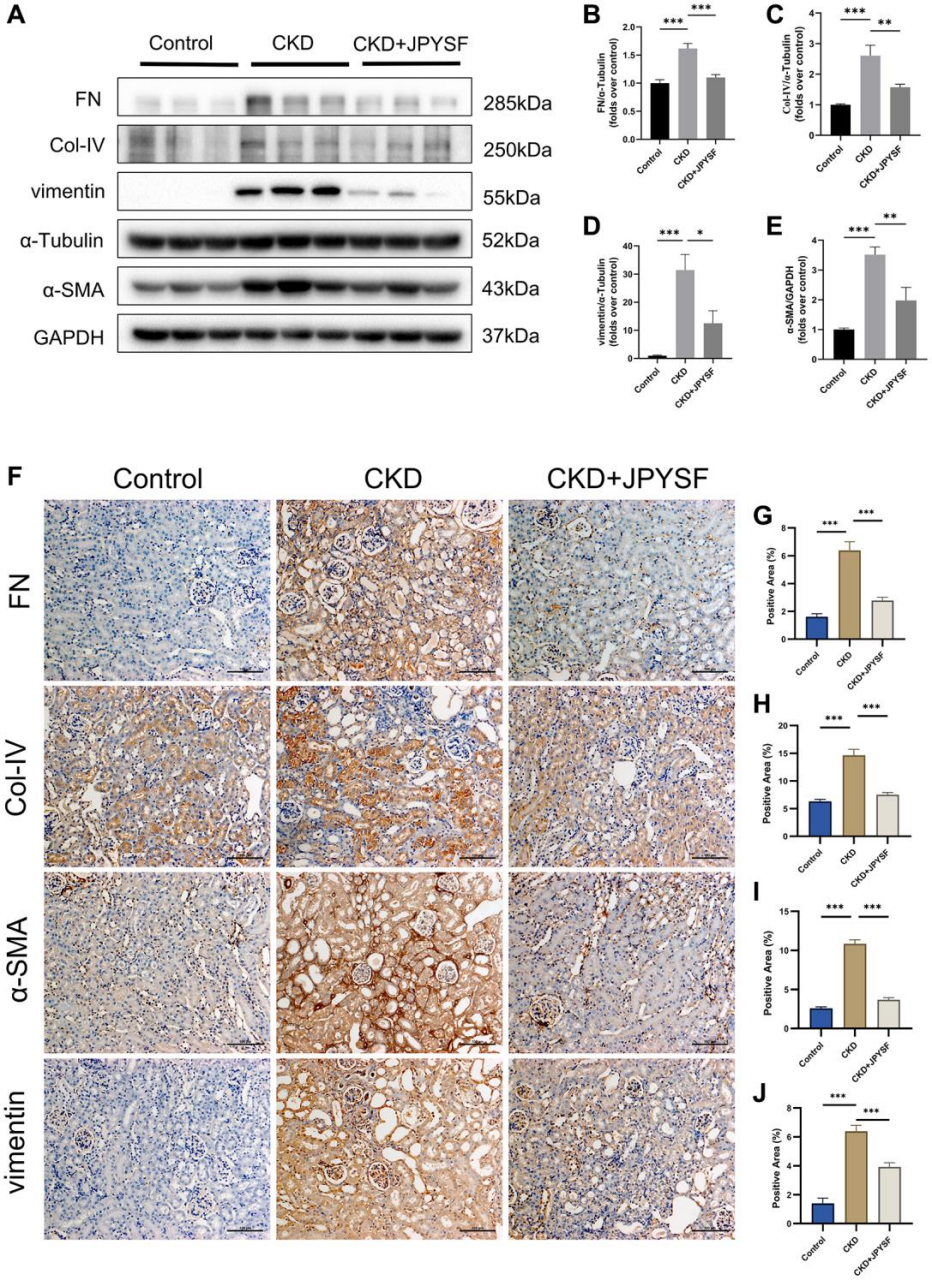
**The effects of JPYSF on renal fibrosis in CKD mice.** (**A**) Representative Western blot images of FN, Col-IV, α-SMA, and vimentin expression in the kidney of mice. (**B**–**E**) Quantitative analysis of FN, Col-IV, α-SMA and vimentin normalized to α-Tubulin or GAPDH content (*n* = 6). (**F**–**J**) Representative immunohistochemical images and quantitative analysis of positive staining areas of FN, Col-IV, α-SMA and vimentin in the kidney of mice (*n* = 3). All images are shown at identical magnification, ×200, scale bar = 100 μm. Data are expressed as mean ± SEM (^*^p < 0.05, ^**^p < 0.01, ^***^p < 0.001 between the indicated two groups).

Overexpression of FN, Col-IV, α-SMA, and vimentin are hallmarks of CKD progression. Western blotting revealed higher levels of FN, Col-IV, α-SMA, and vimentin in the CKD group in contrast to the control group (*p* < 0.001), while administration of JPYSF markedly lowered these proteins expression in CKD mice (*p* < 0.05, Figure 3A–3E). Further results from immunohistochemistry confirmed the inhibitory effect of JPYSF on the expression of fibrosis-associated proteins (Figure 3F–3J). These data indicated that JPYSF could inhibit renal fibrosis in CKD mice.

### JPYSF reversed fibrotic response in TGF-β1-induced HK-2 cells

**Figure 4:**
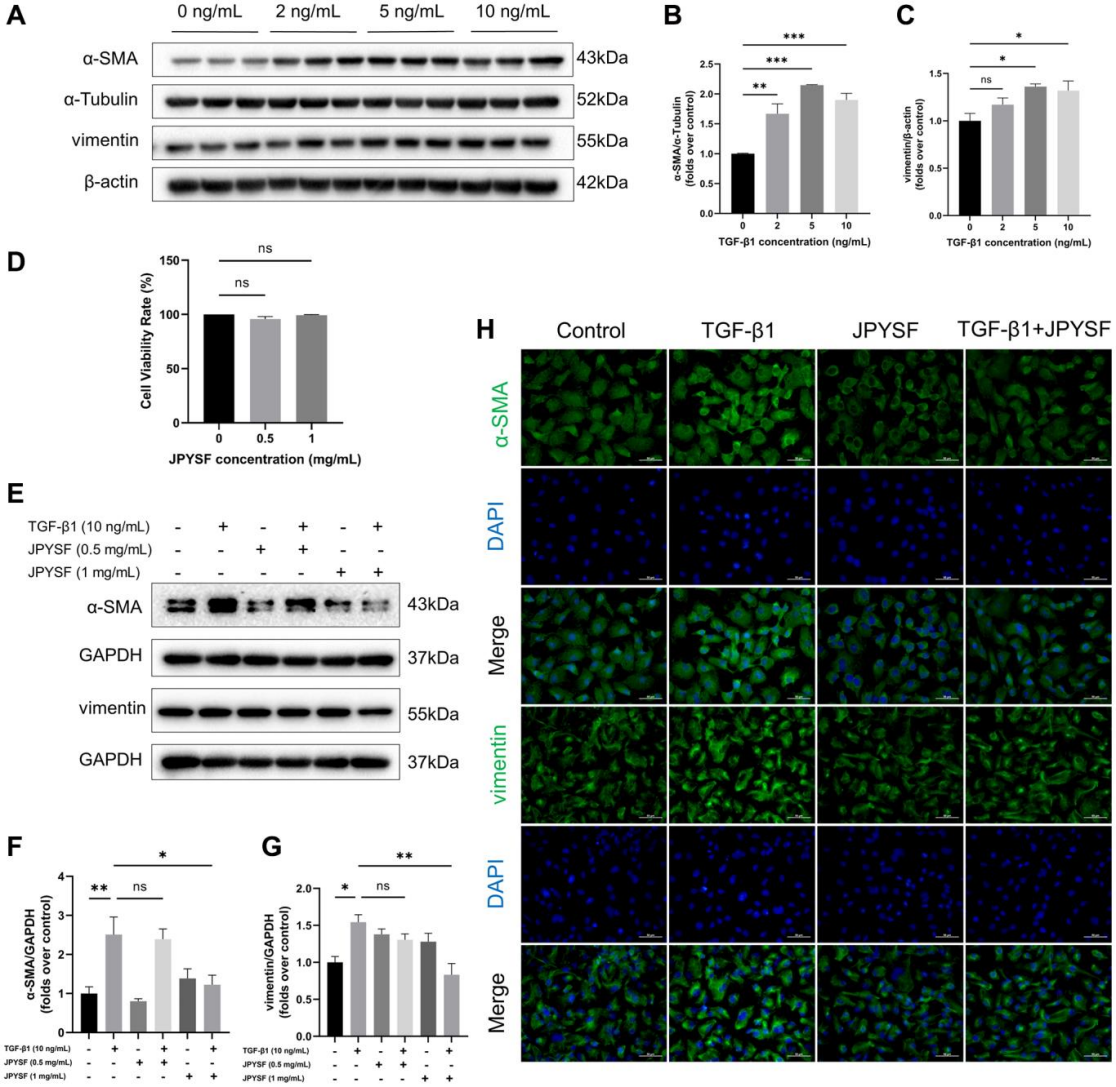
**The effects of JPYSF on fibrotic response in TGF-β1-induced HK-2 cells.** (**A**) Representative Western blot images of α-SMA and vimentin expression in HK-2 cells stimulated with TGF-β1 at the concentrations of 0, 2, 5, and 10 ng/mL. (**B**, **C**) Quantitative analysis of α-SMA and vimentin expression in HK-2 cells stimulated with TGF-β1 at the concentrations of 0, 2, 5, and 10 ng/mL, normalized to α-Tubulin or β-actin content (*n* = 3). (**D**) Cell viability rate (%) (*n* = 3). (**E**) Representative Western blot images of α-SMA and vimentin expression in HK-2 cells stimulated with TGF-β1 or/and JPYSF at the concentrations of 0.5 and 1 mg/mL. (**F**, **G**) Quantitative analysis of α-SMA and vimentin expression in HK-2 cells stimulated with TGF-β1 or/and JPYSF at the concentrations of 0.5 and 1 mg/mL, normalized to GAPDH content (*n* = 3). (**H**) Representative immunofluorescence images of α-SMA and vimentin in HK-2 cells with TGF-β1 or/and 1 mg/mL JPYSF stimulation. Green corresponds to interest proteins, and blue corresponds to nuclear staining. All images are shown at identical magnification, ×400, scale bar = 50 μm. Data are expressed as mean ± SEM (^ns^*p* > 0.05, ^*^p < 0.05, ^**^p < 0.01, ^***^p < 0.001 between the indicated two groups).

As shown in Figure 4A–4C, the expression of fibrosis markers α-SMA and vimentin was elevated in response to TGF-β1 stimulation (*p* < 0.05). JPYSF treatment had no significant effect on HK-2 cell viability (Figure 4D). Western blotting illustrated that 1 mg/mL JPYSF downregulated TGF-β1-induced α-SMA and vimentin expression in HK-2 cells (*p* < 0.05), accompanied by no statistically significant effect at 0.5 mg/mL JPYSF (Figure 4E–4G). The immunofluorescence further confirmed the fact that TGF-β1-induced fibrotic response in HK-2 cells could be obviously reversed by JPYSF (Figure 4H).

### JPYSF rescued the decline in NAD^+^ content in CKD mice and TGF-β1-stimulated HK-2 cells

**Figure 5:**
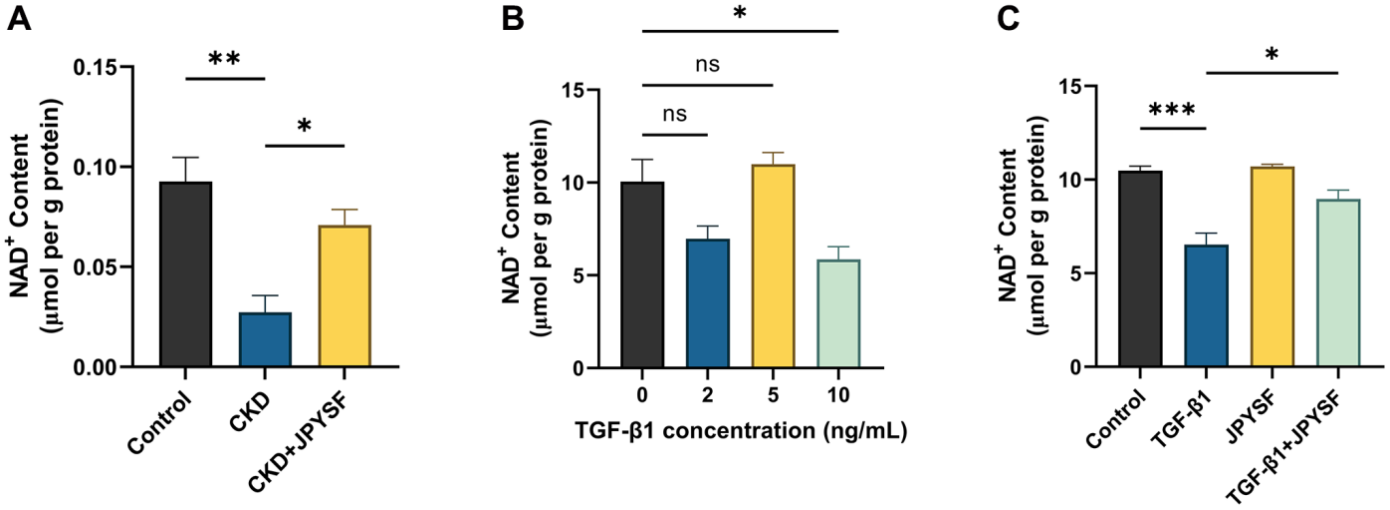
**The effects of JPYSF on NAD^+^ content *in vivo* and *in vitro*.** (**A**) NAD^+^ content in the kidney of normal mice and CKD mice with or without JPYSF treatment (*n* = 4). (**B**) NAD^+^ content in HK-2 cells stimulated with TGF-β1 at the concentrations of 0, 2, 5, and 10 ng/mL (*n* = 3). (**C**) NAD^+^ content in HK-2 cells with TGF-β1 or/and JPYSF stimulation (*n* = 3). Data are expressed as mean ± SEM (^ns^*p* > 0.05, ^*^p < 0.05, ^**^p < 0.01, ^***^p < 0.001 between the indicated two groups).

As exhibited in Figure 5A, there was a substantial reduction in NAD^+^ content in the CKD group compared with the control group (*p* < 0.01), while the decreased NAD^+^ content was rescued by JPYSF treatment (*p* < 0.05). NAD_total_ in the CKD group was significantly lower than that of the control group (*p* < 0.01), and after JPYSF treatment, it increased slightly without statistical significance (Supplementary Figure 1A). In addition, renal NADH content was greatly reduced in CKD mice with or without JPYSF treatment compared with the control group (Supplementary Figure 1B). Similarly, cellular experiments revealed that 10 ng/mL TGF-β1 stimulation of HK-2 cells significantly reduced cellular NAD^+^ content (*p* < 0.05, Figure 5B). JPYSF intervention recovered NAD^+^ content in TGF-β1-stimulated HK-2 cells (*p* < 0.05, Figure 5C). Moreover, 10 ng/mL TGF-β1 stimulation of HK-2 cells most markedly reduced cellular NAD_total_ (*p* < 0.05), while NADH content among all groups changed little (Supplementary Figure 2A, 2B). JPYSF effectively rescued NAD_total_ content but had little effect on NADH (Supplementary Figure 3A, 3B). These results showed that JPYSF could rescue the decline in NAD^+^ content *in vivo* and *in vitro*.

### JPYSF restored NAD^+^ biosynthesis in CKD mice

**Figure 6:**
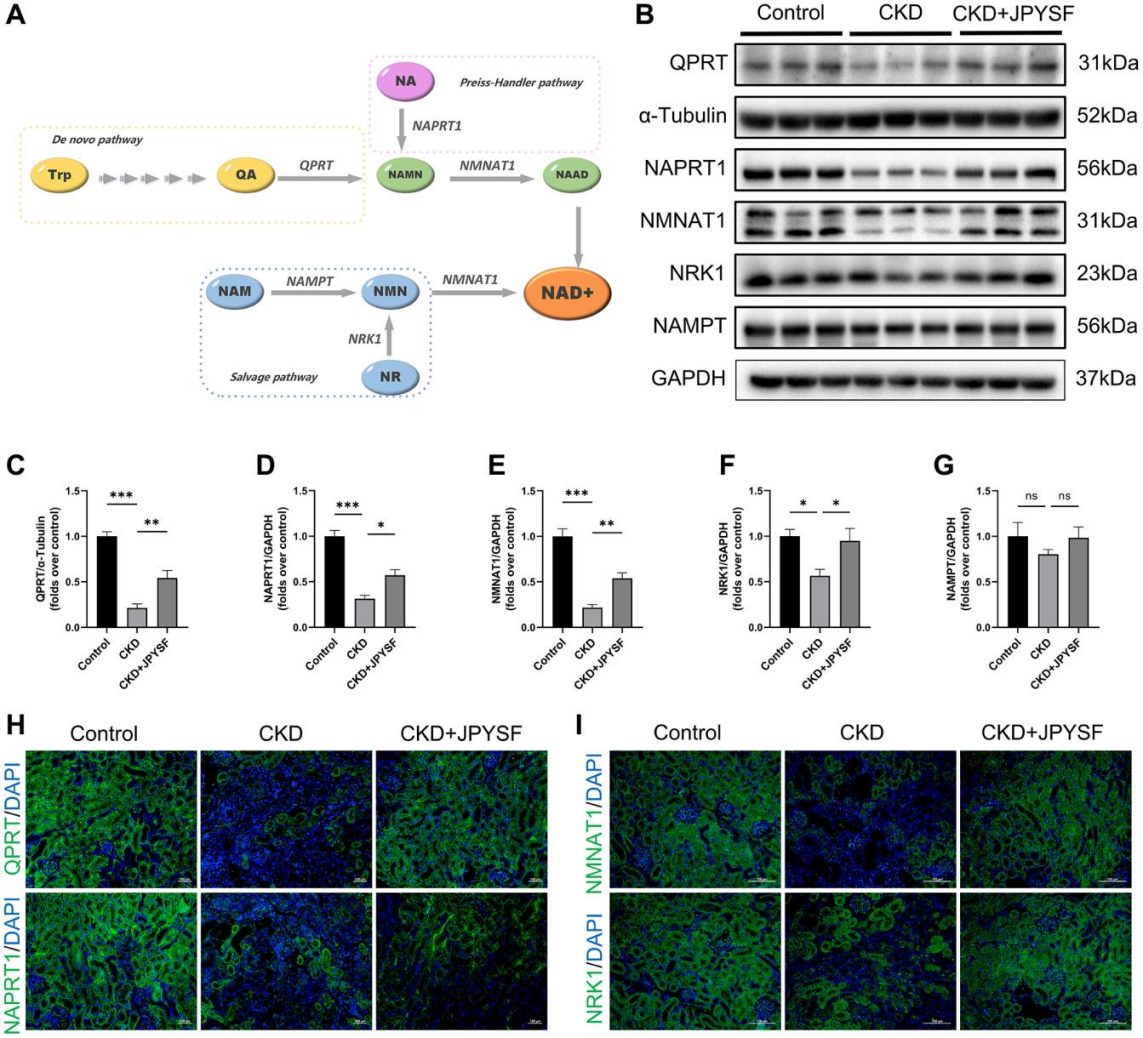
**The effects of JPYSF on the expression of NAD^+^ biosynthesis-related enzymes in mouse kidney.** (**A**) Pathways of NAD^+^ biosynthesis. (**B**) Representative Western blot images of QPRT, NAPRT1, NMNAT1, NRK1, and NAMPT in mouse kidney. (**C**–**G**) Quantitative analysis of QPRT, NAPRT1, NMNAT1, NRK1, and NAMPT expression normalized to α-Tubulin or GAPDH content (*n* = 6). (**H**) Representative immunofluorescence images of QPRT and NAPRT1. (**I**) Representative immunofluorescence images of NMNAT1 and NRK1. Green corresponds to interest proteins, and blue corresponds to nuclear staining. All images are shown at identical magnification, ×200, scale bar = 100 μm. Data are expressed as mean ± SEM (^ns^*p* > 0.05, ^*^p < 0.05, ^**^p < 0.01, ^***^p < 0.001 between the indicated two groups).

NAD^+^ is synthesized through three pathways utilizing various precursors as showed in Figure 6A. To further explore the mechanism by which JPYSF increased NAD^+^ content, we examined the expression levels of five key enzymes involved in NAD^+^ biosynthesis. The gene and protein expression levels of QPRT, NAPRT1, NMNAT1, and NRK1 were down-regulated in the kidney of CKD mice and significantly restored by JPYSF (Supplementary Figure 4, Figure 6B–6F). No statistical difference was observed in NAMPT expression among the groups (Figure 6B, 6G). The results were further confirmed by immunofluorescence staining (Figure 6H, 6I). The findings indicated that NAD^+^ biosynthesis was impaired in the kidney of CKD mice, which could be restored after JPYSF treatment.

### JPYSF restored NAD^+^ biosynthesis in TGF-β1-stimulated HK-2 cells

**Figure 7:**
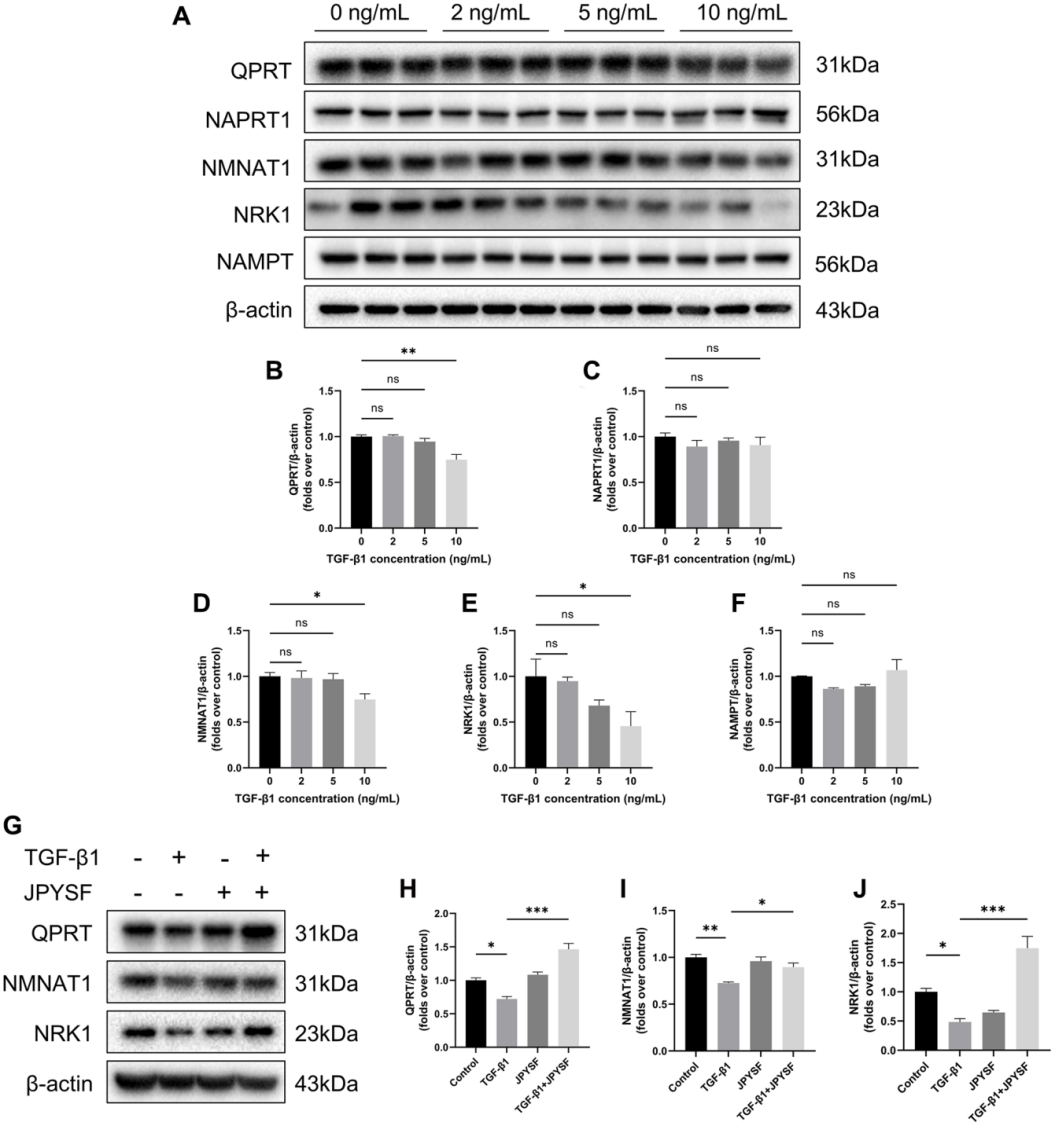
**The effects of JPYSF on the expression of NAD^+^ biosynthesis-related enzymes in HK-2 cells.** (**A**) Representative Western blot images of QPRT, NAPRT1, NMNAT1, NRK1, and NAMPT expression in HK-2 cells stimulated with TGF-β1 at the concentrations of 0, 2, 5, and 10 ng/mL. (**B**–**F**) Quantitative analysis of QPRT, NAPRT1, NMNAT1, NRK1 and NAMPT expression in HK-2 cells stimulated with TGF-β1 at the concentrations of 0, 2, 5 and 10 ng/mL, normalized to β-actin content (*n* = 3). (**G**) Representative Western blot images of QPRT, NMNAT1, and NRK1 expression in HK-2 cells with TGF-β1 or/and JPYSF stimulation. (**H**–**J**) Quantitative analysis of QPRT, NMNAT1, and NRK1 expression in HK-2 cells with TGF-β1 or/and JPYSF stimulation, normalized to β-actin content (*n* = 3). Data are expressed as mean ± SEM (^ns^*p* > 0.05, ^*^p < 0.05, ^**^p < 0.01, ^***^p < 0.001 between the indicated two groups).

Western blotting revealed that the expression of QPRT, NMNAT1, and NRK1 were significantly decreased only in the 10 ng/mL TGF-β1-stimulated group. The expression of NAPRT1 and NAMPT did not change significantly at different concentrations of TGF-β1 stimulation (Figure 7A–7F). Therefore, we further questioned whether JPYSF could influence the expression of QPRT, NMNAT1, and NRK1 in HK-2 cells. Although there were no significant differences between groups at mRNA level, the addition of JPYSF intervention significantly restored the protein expression of the above three enzymes compared with the TGF-β1 group (Supplementary Figure 5, Figure 7G–7J. Primer sequences for qPCR were listed in Supplementary Table 1). These data suggested that TGF-β1 interfered with NAD^+^ biosynthesis in HK-2 cells, which could be corrected by JPYSF.

## DISCUSSION

In this study, we examined the effects of JPYSF on renal fibrosis and NAD^+^ biosynthesis in CKD mouse models induced by adenine, as well as HK-2 cells stimulated by TGF-β1. We found that JPYSF down-regulated fibrotic proteins expression and restored NAD^+^ content both *in vivo* and *in vitro*. What’s more, JPYSF treatment reversed the down-regulation of key enzymes (QPRT, NAPRT1, NMNAT1, and NRK1) involved in NAD^+^ biosynthesis in CKD mice. Similarly, stimulation with 10 ng/mL TGF-β1 significantly reduced QPRT, NMNAT1, and NRK1 expression in HK-2 cells, which was inhibited by JPYSF. In contrast, the expression of NAMPT did not differ significantly among groups both *in vivo* and *in vitro*.

Beyond its central role in oxidation-reduction reactions for energy harvesting, NAD^+^ is an essential co-substrate in many enzymatic reactions serving different functions. Therefore, NAD^+^ deficiency is implicated in the progression of various diseases, including aging, neurodegeneration, and cancer [15, 27, 33–35]. The fall in renal NAD^+^ levels reduces the respiratory function of mitochondria, which can lead to mitochondrial dysfunction and reactive oxygen species (ROS) accumulation [36]. As a result, the highly energy-dependent renal tubular epithelial cells would fail in filtration and reabsorption, leading to impaired renal function [22]. NAD^+^ levels have been reported to be significantly decreased in Ischaemia-reperfusion injury (IRI)-induced AKI mice [24, 37], cisplatin-induced AKI mice [37], and ischemic human kidney [24]. Although Zheng et al. reported that NAM reduced renal fibrosis in unilateral ureteral obstruction (UUO) mice model and TGF-β stimulated mouse proximal tubule cells, changes in NAD^+^ content *in vivo* and *in vitro* were not clear [38]. In CKD models, declined renal NAD^+^ levels have been reported by Kumakura et al. and our group [28, 39]. In our present study, we further confirmed the decrease of NAD^+^ content in the kidney of adenine-induced CKD mice. Moreover, cellular NAD^+^ content was also decreased during TGF-β1-induced pro-fibrotic changes in proximal tubular epithelial cells. This implies that NAD^+^ deficiency may contribute to the process of CKD and renal fibrosis.

Although impaired NAD^+^ synthesis has been found to be a marker of CKD [25, 28], it is puzzling that administration of NAD^+^ boosters have shown different outcomes in preventing CKD progression in animals and patients [22]. Several studies have focused on boosting the NAD^+^ salvage pathway. NAM in the salvage pathway is catalyzed by NAMPT to generate NMN and eventually NAD^+^ [22]. Although NAM supplementations have been shown to prevent CKD progression in preclinical models by improving renal dysfunction and fibrosis [38–41], a clinical trial reported that boosting NAD^+^ by NAM supplementation did not have a significant effect in CKD patients [42]. Moreover, the major metabolite of NAM, N-Me-2PY, has been found to accumulate in serum during NAM treatment. N-Me-2PY was reported to be a uremic toxin and can cause renal cell damage [43, 44]. Our results showed that NAMPT expression was not statistically different among all groups both *in vivo* and *in vitro*. This may be one of the reasons why the aforementioned NAM supplementation could not be fully utilized, leading to N-Me-2PY accumulation. In the salvage pathway, NR is catalyzed by NRKs to generate NMN and subsequently NAD^+^. Since non-vasoactive NR does not induce flushing, NR has emerged as a promising alternative NAD^+^ precursor and available NR chloride supplement have been approved in 2013 [15, 45]. Although it has shown preclinical potential to treat AKI, NR has failed to prevent CKD progression in animal models [25, 46]. Our results showed that NRK1 was significantly decreased in CKD model and TGF-β1-induced HK-2 cells, which probably indicated that research should focus more on NRK1 under the strong application potential of NR in CKD. In the Preiss-Handler pathway, our result showed that the expression of NAPRT1 varied *in vivo* but not *in vitro*, which may be related to the complicated metabolic mechanism *in vivo*. Although NA has been identified to improve inflammation, proteinuria, hypertension, tubular injury, and oxidative stress in 5/6 nephrectomy model, it has no significant effect on reducing serum creatinine [47]. Moreover, studies have reported that the *de novo* pathway was damaged in cases of AKI and a persistent reduction of QPRT expression accelerated the progression from AKI to CKD [24, 28, 48, 49]. Even though tryptophan is the precursor of the *de novo* pathway, it has been identified that the alteration of tryptophan metabolism in CKD leaded to the release of uremic toxins [50]. Moreover, for the reason that tryptophan also serves as a precursor to neurotransmitters and other signaling proteins, its low conversion ratio makes it an inadequate precursor for synthesizing NAD^+^ in CKD [15]. In our study, we observed a significant downregulation of QPRT in CKD mice and TGF-β1-stimulated HK-2 cells. This implies that targeting QPRT to increase its expression may be an effective way to boost NAD^+^ production and thus delay CKD progression.

Traditional Chinese medicine has been widely used in the treatment of CKD. As a basic prescription for patients with CKD, JPYSF has a good efficiency on CKD with over 20 years of clinical verification [51]. Renal fibrosis is a common terminal outcome of CKD, leading to progressive loss of renal function and eventually kidney failure. Studies have shown that JPYSF protected kidney from fibrotic injury [12, 13, 51]. Some active ingredients in JPYSF have been reported to exert anti-fibrotic effects through acting on cellular signal pathways, such as inhibiting the TGF-β1 pathway, activating Sirt1-mediated autophagy, and inhibiting the Wnt3a/β-catenin pathway [52–56]. Our data suggested that JPYSF alleviates renal fibrosis by restoring NAD^+^ biosynthesis. Notably, JPYSF upregulated the expression of several key enzymes for NAD^+^ synthesis. This observation not only reflects the characteristics of multi-target regulation of traditional Chinese medicine, but also provides a direction for further mechanism exploration. Till now, several components existed in JPYSF have been reported to boost NAD^+^ content for therapeutic effects. Emodin, a natural anthraquinone compound existed in Rhei Radix et Rhizoma (Da Huang), prevented the depletion of intracellular NAD^+^ and ATP induced by Zn^2+^ overload in neurons [57]. Salvianolic acid B could reverse cellular NAD^+^ depletion induced by angiotensin II to protect cardiomyocyte [58]. A protective effect of quercetin on mitochondrial function under high glucose-induced stress in HepG2 cells was found to be attributed to an increase in NAD^+^ content and the activation of PGC-1α-mediated pathways [59]. However, the specific components that play a role and their corresponding targets need further exploration.

## CONCLUSIONS

In conclusion, JPYSF alleviated renal fibrosis in adenine-induced CKD mice and attenuated the fibrotic response in TGF-β1-stimulated HK-2 cells, potentially through restoration of NAD^+^ biosynthesis. This study provided a new insight into the mechanism of JPYSF against CKD and suggested that NAD^+^ biosynthesis might be a promising therapeutic target in treating fibrotic renal disease.

## Supplementary Materials

Supplementary Materials

Supplementary Figures

Supplementary Table 1
