# Supplementary material for: Jian-Pi-Yi-Shen formula alleviates renal fibrosis by restoring NAD+ biosynthesis in vivo and in vitro
Source: Aging (Albany NY). 2023 Dec 28;16(1):106–28. doi: 10.18632/aging.205352 (PMC10817388; doi:10.18632/aging.205352)
Supplement: Supplementary Materials [file aging-16-205352-s001.pdf]

SUPPLEMENTARY MATERIALS

JPYSF preparation and extraction

Dry sample extraction

Using vacuum freeze-drying technology, the biological samples were placed in a lyophilizer (Scientz-100F), then grinding (30 Hz, 1.5 min) the samples to powder form by using a grinder (MM 400, Retsch). Next, weigh 50 mg of sample powder using an electronic balance (MS105DM) and add 1200 μL of −20°C pre-cooled 70% methanolic aqueous internal standard extract (less than 50 mg added at the rate of 1200 μL extractant per 50 mg sample). Vortex once every 30 min for 30 sec, for a total of 6 times. After centrifugation (rotation speed 12000 rpm, 3 min), the supernatant was aspirated, and the sample was filtered through a microporous membrane (0.22 μm pore size) and stored in the injection vial for UPLC-MS/MS analysis.

UPLC conditions

The sample extracts were analyzed using an UPLC-ESI-MS/MS system (UPLC, ExionLC™ AD, https://sciex.com.cn/; MS, Applied Biosystems 4500 Q TRAP, https://sciex.com.cn/). The analytical conditions were as follows, UPLC: column, Agilent SB-C18 (1.8 μm, 2.1 mm × 100 mm); The mobile phase consisted of solvent A, pure water with 0.1% formic acid, and solvent B, acetonitrile with 0.1% formic acid.

Sample measurements were performed with a gradient program that employed the starting conditions of 95% A, 5% B. Within 9 min, a linear gradient to 5% A, 95% B was programmed, and a composition of 5% A, 95% B was kept for 1 min. Subsequently, a composition of 95% A, 5.0% B was adjusted within 1.1 min and kept for 2.9 min. The flow velocity was set as 0.35 mL per minute; The column oven was set to 40°C; The injection volume was 4 μL. The effluent was alternatively connected to an ESI-triple quadrupole-linear ion trap (QTRAP)-MS.

ESI-Q TRAP-MS/MS

The ESI source operation parameters were as follows: source temperature 550°C; ion spray voltage (IS) 5500 V (positive ion mode)/-4500 V (negative ion mode); ion source gas I (GSI), gas II (GSII), curtain gas (CUR) were set at 50, 60, and 25 psi, respectively; the collision-activated dissociation (CAD) was high. QQQ scans were acquired as MRM experiments with collision gas (nitrogen) set to medium. DP (declustering potential) and CE (collision energy) for individual MRM transitions was done with further DP and CE optimization. A specific set of MRM transitions were monitored for each period according to the metabolites eluted within this period.
