# Supplementary material for: Jian-Pi-Yi-Shen formula alleviates renal fibrosis by restoring NAD+ biosynthesis in vivo and in vitro
Source: Aging (Albany NY). 2023 Dec 28;16(1):106–28. doi: 10.18632/aging.205352 (PMC10817388; doi:10.18632/aging.205352)
Supplement: Supplementary Figures [file aging-16-205352-s002.pdf]

SUPPLEMENTARY FIGURES

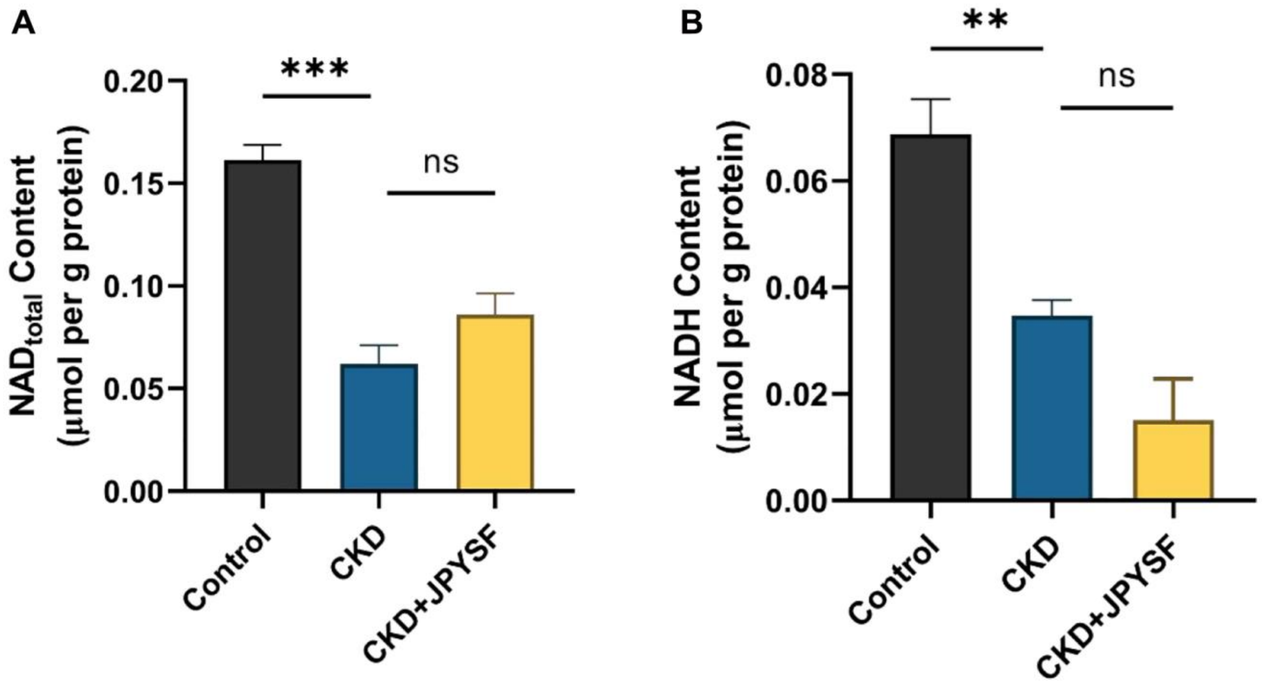

Supplementary Figure 1. NAD$_{total}$ and NADH content in the kidney of normal mice and CKD mice with or without JPYSF treatment. (**A**) NAD$_{total}$ content (n = 4). (**B**) NADH content (n = 4). Data are expressed as mean ± SEM ($^{**}p < 0.01$, $^{***}p < 0.001$ between the indicated two groups).

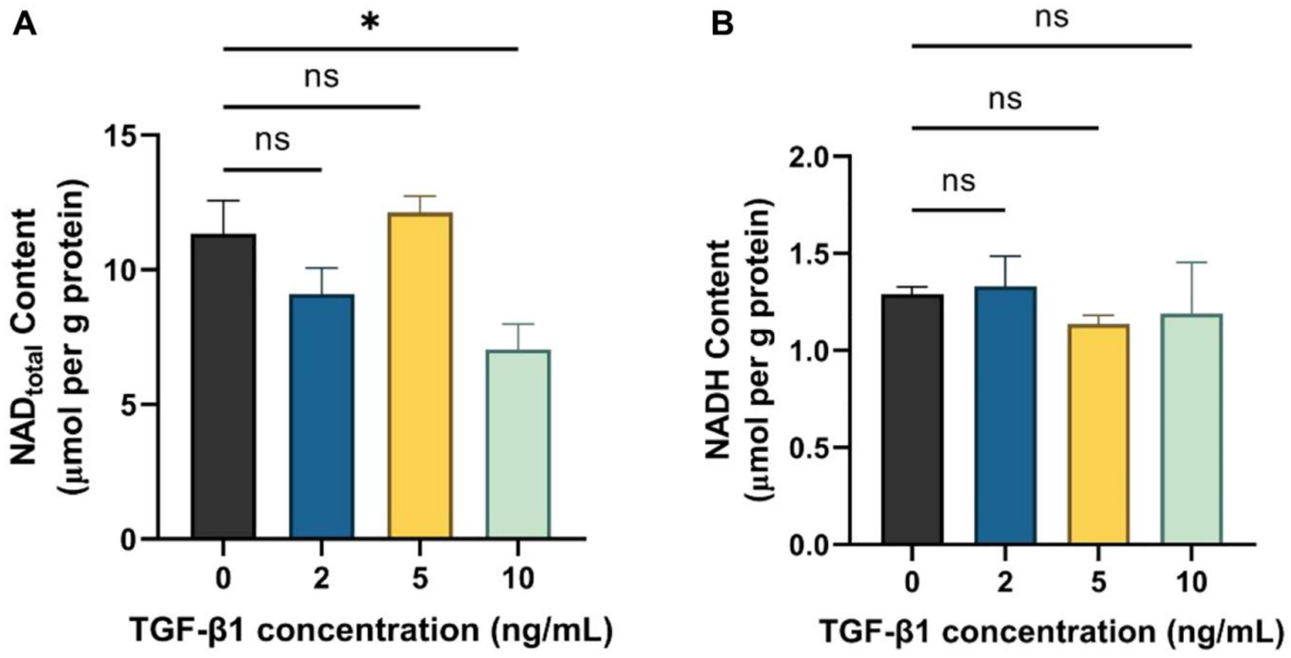

Supplementary Figure 2. NAD$_{total}$ and NADH content in HK-2 cells stimulated with TGF-β1 at the concentrations of 0, 2, 5, and 10 ng/mL. (**A**) NAD$_{total}$ content (n = 3). (**B**) NADH content (n = 3). Data are expressed as mean ± SEM ($^{*}p < 0.05$ between the indicated two groups).

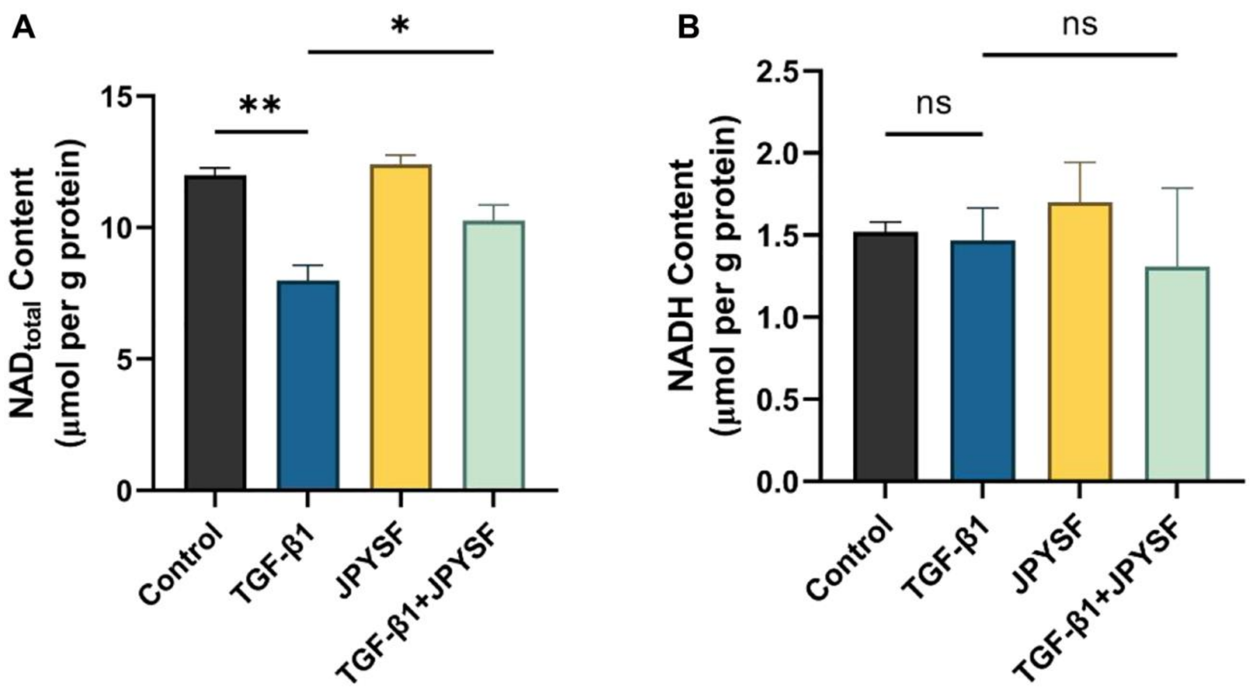

Supplementary Figure 3. NAD_{total} and NADH content in HK-2 cells with TGF-β1 or/and JPYSF stimulation. (**A**) NAD_{total} content (n = 3). (**B**) NADH content (n = 3). Data are expressed as mean ± SEM ($^{*}p < 0.05$ between the indicated two groups).

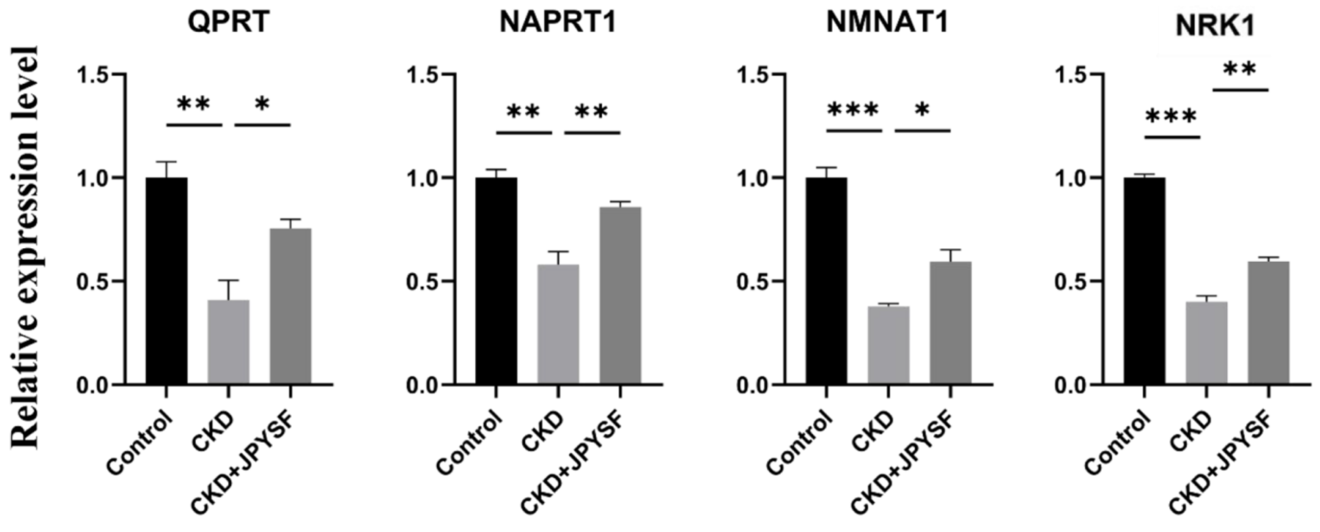

Supplementary Figure 4. NAD^+ biosynthesis-related enzymes in mouse kidney were analyzed by qPCR (n = 3). Data are expressed as mean ± SEM ($^{*}p < 0.05$, $^{**}p < 0.01$, $^{***}p < 0.001$ between the indicated two groups).

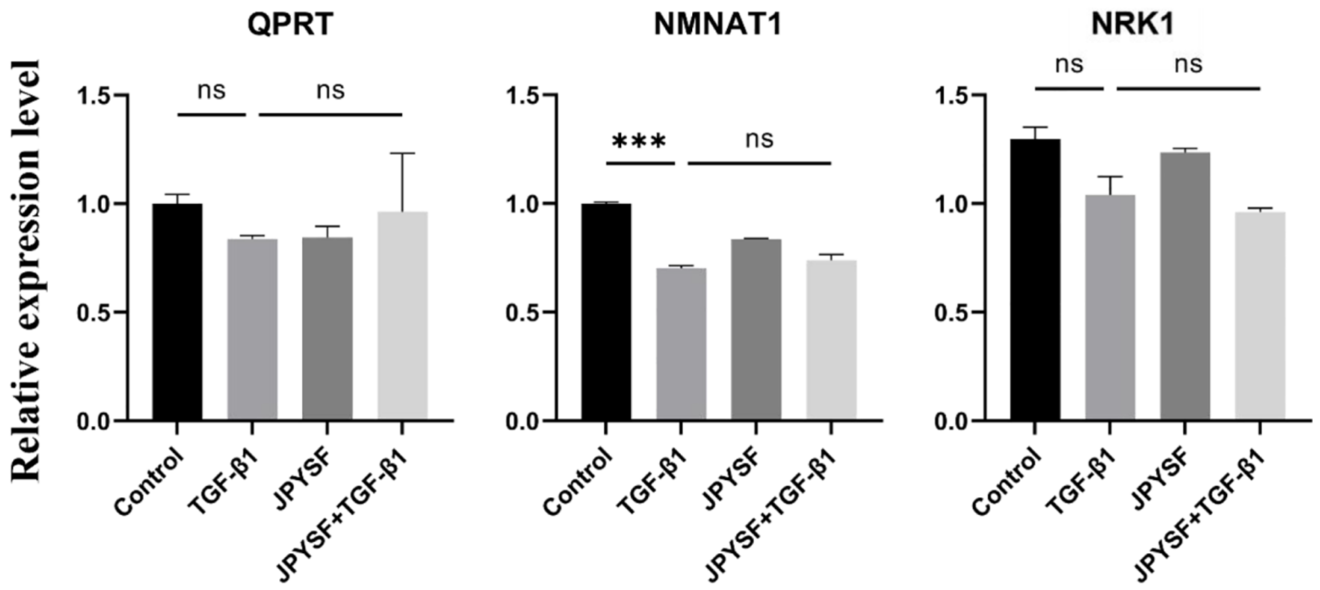

Supplementary Figure 5. NAD$^+$ biosynthesis-related enzymes in HK-2 cells with TGF-β1 or/and JPYSF stimulation were analyzed by qPCR (*n* = 2~3). Data are expressed as mean ± SEM ($^{***}p < 0.001$ between the indicated two groups).
