# Supplementary material for: Jian-Pi-Yi-Shen formula alleviates renal fibrosis by restoring NAD+ biosynthesis in vivo and in vitro
Source: Aging (Albany NY). 2023 Dec 28;16(1):106–28. doi: 10.18632/aging.205352 (PMC10817388; doi:10.18632/aging.205352)
Supplement: Supplementary Table 1 [file aging-16-205352-s003.pdf]

SUPPLEMENTARY TABLE

Supplementary Table 1. Primer sequences for qPCR analysis.

	Target	Primer sequence (5′ → 3′)
m-QPRT	Forward	CCGGGCCTCAATTTTGCATC
	Reverse	GGTGTTAAGAGCCACCCGTT
m-NAPRT1	Forward	TGCTCACCGACCTCTATCAGG
	Reverse	GCGAAGGAGCCTCCGAAAG
m-NMNAT1	Forward	CGGTCGGTGATGCGTACAAC
	Reverse	TCCAGTGCAGGTGAGCTTTG
m-NRK1	Forward	TCATTGGAATTGGTGGTGTGAC
	Reverse	CAACAGGAAACTGCTGACATCAT
m-Actin	Forward	GAGACCTTCAACACCCCAGC
	Reverse	ATGTCACGCACGATTTCCC
h-QPRT	Forward	GGGCAGCCTTTCTTCGATG
	Reverse	GGAGCCCATACTTCTCCACCA
h-NMNAT1	Forward	TCTCCTTGCTTGTGGTTCATTC
	Reverse	TGACAACTGTGTACCTTCCTGTT
h-NRK1	Forward	TCAGTGGTGTGACAAACAGTG
	Reverse	GCACATCGTACTGCAAAAATCC
h-Actin	Forward	TGACGTGGACATCCGCAAAG
	Reverse	CTGGAAGGTGGACAGCGAGG

Abbreviations: m: mouse; h: human.
